# Synergistic Effects on the Elderly People's Motor Control by Wearable Skin-Stretch Device Combined with Haptic Joystick

**DOI:** 10.3389/fnbot.2017.00031

**Published:** 2017-06-23

**Authors:** Han U. Yoon, Namita Anil Kumar, Pilwon Hur

**Affiliations:** Department of Mechanical Engineering, Texas A&M UniversityCollege Station, TX, United States

**Keywords:** sensory feedback combination, force feedback, skin-stretch feedback, haptic joystick, wearable skin-stretch device, motor control improvement

## Abstract

Cutaneous sensory feedback can be used to provide additional sensory cues to a person performing a motor task where vision is a dominant feedback signal. A haptic joystick has been widely used to guide a user by providing force feedback. However, the benefit of providing force feedback is still debatable due to performance dependency on factors such as the user's skill-level, task difficulty. Meanwhile, recent studies have shown the feasibility of improving a motor task performance by providing skin-stretch feedback. Therefore, a combination of two aforementioned feedback types is deemed to be promising to promote synergistic effects to consistently improve the person's motor performance. In this study, we aimed at identifying the effect of the combined haptic and skin-stretch feedbacks on the aged person's driving motor performance. For the experiment, 15 healthy elderly subjects (age 72.8 ± 6.6 years) were recruited and were instructed to drive a virtual power-wheelchair through four different courses with obstacles. Four augmented sensory feedback conditions were tested: no feedback, force feedback, skin-stretch feedback, and a combination of both force and skin-stretch feedbacks. While the haptic force was provided to the hand by the joystick, the skin-stretch was provided to the steering forearm by a custom-designed wearable skin-stretch device. We tested two hypotheses: (i) an elderly individual's motor control would benefit from receiving information about a desired trajectory from multiple sensory feedback sources, and (ii) the benefit does not depend on task difficulty. Various metrics related to skills and safety were used to evaluate the control performance. Repeated measure ANOVA was performed for those metrics with two factors: task scenario and the type of the augmented sensory feedback. The results revealed that elderly subjects' control performance significantly improved when the combined feedback of both haptic force and skin-stretch feedback was applied. The proposed approach suggest the feasibility to improve people's task performance by the synergistic effects of multiple augmented sensory feedback modalities.

## 1. Introduction

For the past one decade, augmentation of sensory feedback has been widely used to improve peoples' task performance related to the activities of daily living (Sigrist et al., 2013). Recent studies have reported that people's task performance can be improved by applying various types of augmented sensory feedback such as visual feedback (Blandin et al., 2008; Huet et al., 2009; Ranganathan and Newell, 2009; Snodgrass et al., 2010; Sülzenbrück and Heuer, 2011; Franz et al., 2014), auditory feedback (Riskowski et al., 2009; Eriksson et al., 2011; Helmer et al., 2011; Secoli et al., 2011; Sigrist et al., 2011), haptic feedback (Reinkensmeyer and Patton, 2009; Chen et al., 2011; Powell and O'Malley, 2011; Marchal-Crespo et al., 2012), and multimodal feedback (Ronsse et al., 2011; Wang et al., 2011; Sigrist et al., 2015). There exist various approaches and practical applications related to the augmented sensory feedbacks. In these studies, the augmented sensory feedbacks were often referred to by several interchangeable names, e.g., augmented feedback, biofeedback, extrinsic feedback (Schmidt and Wrisberg, 2008; Utley and Astill, 2008).

Among the types of the introduced augmented sensory feedback, haptic feedback is the one that can deliver tangible feedbacks, e.g., force, stretch, or vibration, to users' body parts. A haptic lever is one of the most commonly-used interfaces providing a force feedback to attract the user toward the safer or the desired directions while driving a power-wheelchair (Crespo and Reinkensmeyer, 2008; Marchal-Crespo et al., 2010a; Yoon et al., 2017) or assisting target-pointing/hitting tasks (Powell and O'Malley, 2012; Fisher et al., 2015; Patton and Huang, 2016). Vibrators or skin-stretchers have also been used to provide tactile stimulations for postural sway improvement (Gopalai and Senanayake, 2011; Pan and Hur, 2016; Pan et al., 2017), trunk sway improvement (Davis et al., 2010; Lee et al., 2012), target acquisition and pointing (Bark et al., 2010; Hsieh et al., 2014; Kaul and Rohs, 2016), balance training (Spelmezan et al., 2009; Nanhoe-Mahabier et al., 2012), gait function learning (Shull et al., 2011; Sienko et al., 2013). Several devices have been developed to provide realistic three dimensional tactile sensation to the user, e.g., touching a flat surface, grasping a virtual object, and tipping a surface or an object (Chinello et al., 2012; Prattichizzo et al., 2013; Pacchierotti et al., 2015).

Although there exist many investigations about the effect of haptic sensory augmentation, the benefit from the haptic sensory augmentation is still debatable. It has been known that the performance improvement under haptic guidance depends on the user's age and initial skill level (Marchal-Crespo et al., 2010b). Specifically, haptic guidance is more suitable for the novice subjects than the skilled subjects, and the significant improvement could be observed mostly for the for young adults compared to the elderly adults (Cesqui et al., 2008; Milot et al., 2010; Marchal-Crespo et al., 2010b). A critical drawback of the haptic guidance that has been reported is that the haptic guidance can improve the user's performance mostly for the simple motor tasks, e.g., pointing or reaching tasks; in contrast, its benefits tend to degrade for more complex motor tasks, e.g., driving tasks, learning motions in sport activity (Todorov, 2004; Rauter et al., 2010; Marchal-Crespo et al., 2012). All of these issues have been observed in our previous research where subjects drove a virtual power-wheelchair without colliding against obstacles under haptic guidance (Yoon and Hur, 2016). Considering that elderly adults are usually novice drivers, the observed inconsistency of performance improvement across the elderly adults impeded extending our methods for power-wheelchair guidance to the elderly adults.

To overcome these issues and advance state-of-the-arts in both multiple haptic modalities and haptic guidance for the elderly adults, we propose a haptic joystick combined with a custom-designed wearable skin-stretcher to more effectively guide the elderly adults while driving a power-wheelchair. The objectives of this study are (i) to examine the synergistic effects between two haptic sensory feedback modalities, i.e., force feedback to the hand and cutaneous skin stretch feedback on the steering forearm, (ii) and observe if the synergistic effects are consistent throughout various scenarios. Our hypothesis is that these two simultaneous feedbacks can provide more reliable and informative sensory cues for the improved driving performance in the elderly population. This is plausible because the additional skin stretch feedback can provide more reliable guidance on the supination/pronation of the forearm, which plays an important role in the rider's power-wheelchair control. To the best of authors' knowledge, combining force feedback and cutaneous skin stretch feedback for elderly subjects to improve power-wheelchair control has not been considered.

To validate the proposed approach, an experiment with the elderly subjects was performed. In the experiment, the subjects were exposed to four different task scenarios, and their performance was recorded under four different conditions of the augmented sensory feedback: no feedback augmentation, force feedback augmentation by a haptic joystick, cutaneous skin stretch augmentation by a wearable skin-stretcher, and a combination of force feedback and cutaneous skin stretch by the two devices. Statistical analyses were performed to identify the effects of two factors: task scenarios and augmented sensory feedback. The rest of the paper is organized as follows. In Section 2, the materials and method for this study are introduced. Section 3 describes the human subject experiment. Results are presented in Section 4. Discussions related to findings throughout this study are provided in Section 5.

## 2. Materials and methods

### 2.1. Assistive power-wheelchair simulator for the elderly riders: system overview

**Figure 1:**
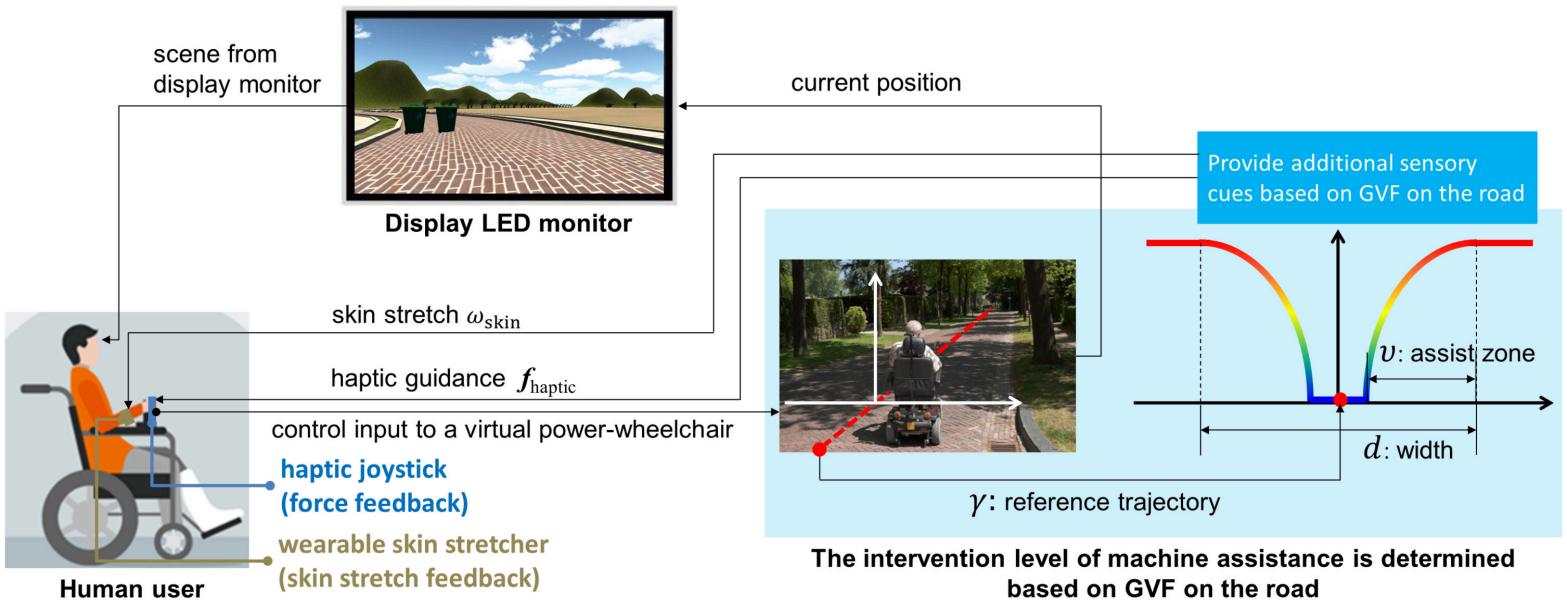
The architecture of an assistive power-wheelchair simulator.

Figure 1 depicts the architecture of the developed assistive power-wheelchair simulator. A 2D haptic interface and a wearable skin-stretcher were custom-developed to guide a rider (subject) by providing force feedback to the hand and skin stretch feedback on the forearm, respectively. The anatomies of the two developed sensory augmentation devices are further discussed in Section 2.2. To assist the rider effectively, the intensity and the direction of these augmented sensory feedbacks should be adjusted in real time according to the quality of the subject's control. To achieve this, we adopt a guidance-based virtual fixture (GVF) which sets a monotonically non-increasing function $\kappa_{\delta^{⊥}}$ on the given reference trajectory γ. The reference trajectory is an artificially-generated trajectory by a machine intelligence, within a width *d* (Bettini et al., 2004; Abbott et al., 2007). We will discuss how to dynamically adjust the augmented sensory feedbacks in Section 2.3.

**Figure 2:**
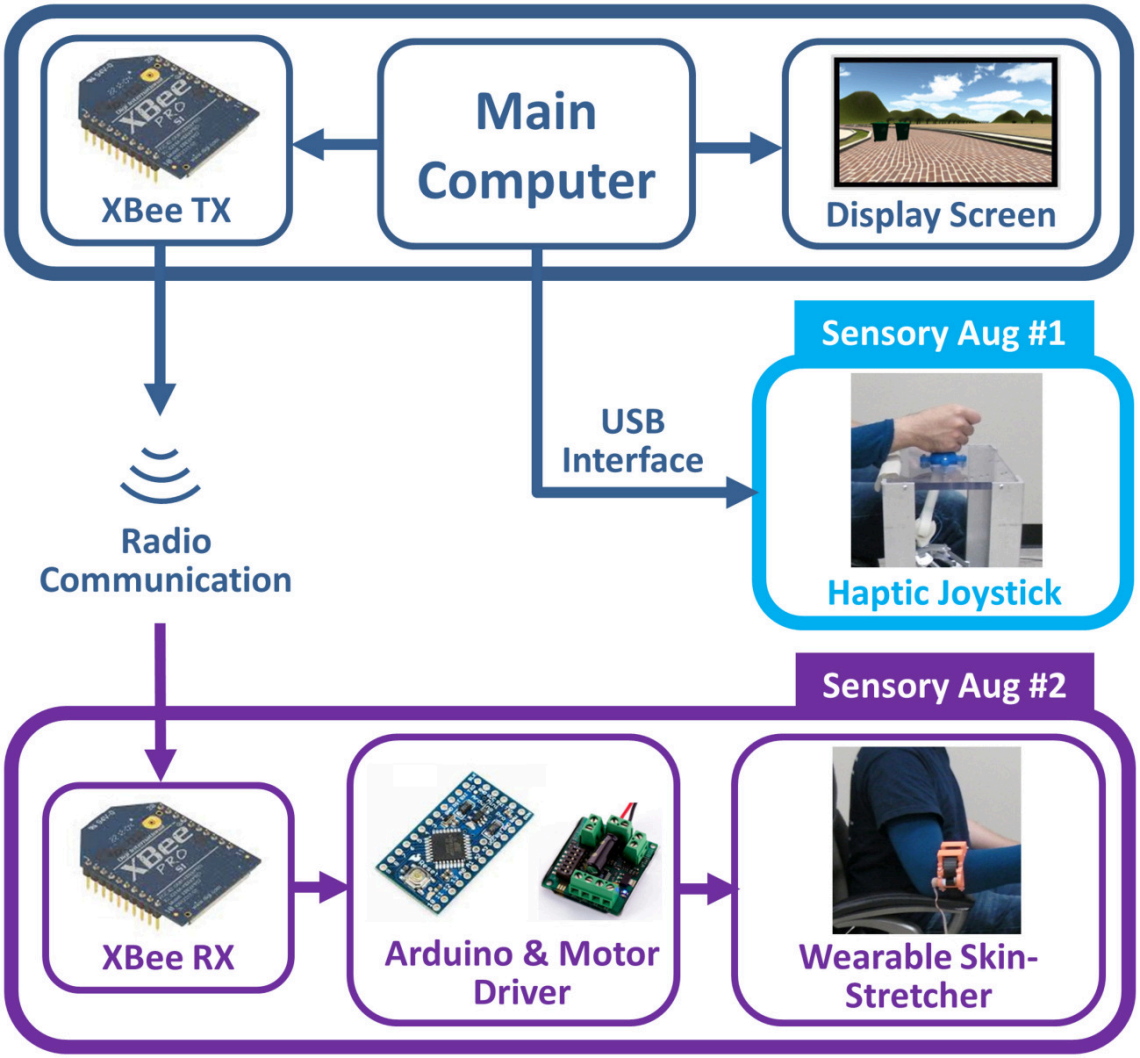
Experimental system apparatus for human subject experiment: The augmented sensory feedback condition was corresponded to the two feedbacks via the haptic joystick or the wearable skin-stretcher.

Figure 2 illustrates our experimental system apparatus in which a main computer displays environmental scene and controls two sensory augmentation devices. The main computer communicated with the haptic joystick via USB interface to generate a force feedback. The control signal for the wearable skin-stretcher was transmitted via XBee radio communication (XBP24-API-001, Digi International, Minnetonka, MN) so that a rider would not be tethered to the power-wheelchair by a wiring between the skin-stretcher and the power-wheelchair. The received control signal was sent to the micro-controller (Arduino Mini 5V, Adafruit Industries, New York, NY) + motor driver (Sabertooth 2 x 5, Dimension Engineering, Akron, OH) to actuate the wearable skin-stretcher. Note that, for human subject experiments, the augmented sensory feedback condition was corresponded to which the subject was provided with the two sensory feedbacks via the haptic joystick or the wearable skin-stretcher. Although the visual display is also a sensory signal, the visual display was not considered/manipulated for the sensory augmentations in this study.

### 2.2. Wearable skin-stretcher and haptic joystick to provide two augmented sensory feedbacks

**Figure 3:**
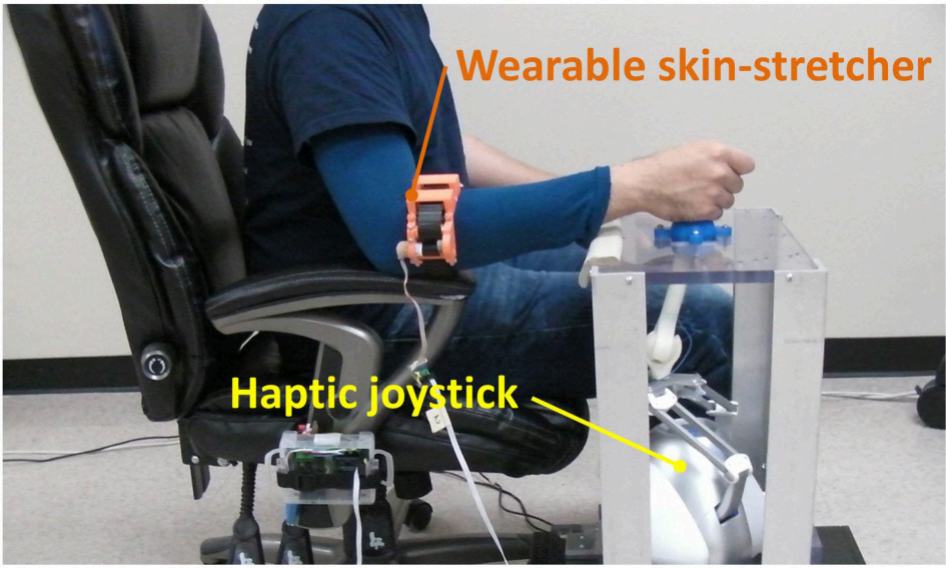
Custom-developed two sensory augmentation devices: the wearable skin-stretcher mounted on a subject's forearm and the 2D haptic joystick.

Our custom-designed wearable skin-stretcher and a 2D haptic joystick were developed to provide two augmented sensory feedbacks to the subject. Figure 3 shows the wearable skin-stretcher worn on the subject's forearm, and the 2D haptic joystick being manipulated by the subject's hand. While the haptic joystick can be manipulated by the subject, it can also provide a force feedback to the subject. The spot for the skin-stretcher on the subject's forearm was determined based on findings from our preliminary research (Yoon et al., 2016). Suppose that the subject is wearing the skin-stretcher on the right forearm. Then the mounted spot can be stretched toward left (i.e., pronation) or right (i.e., supination) by the clockwise/counterclockwise rotation of a timing belt. Simultaneously, the subject's manipulating hand is pulled or pushed by the lever of the haptic joystick in the same direction as the skin stretch.

**Figure 4:**
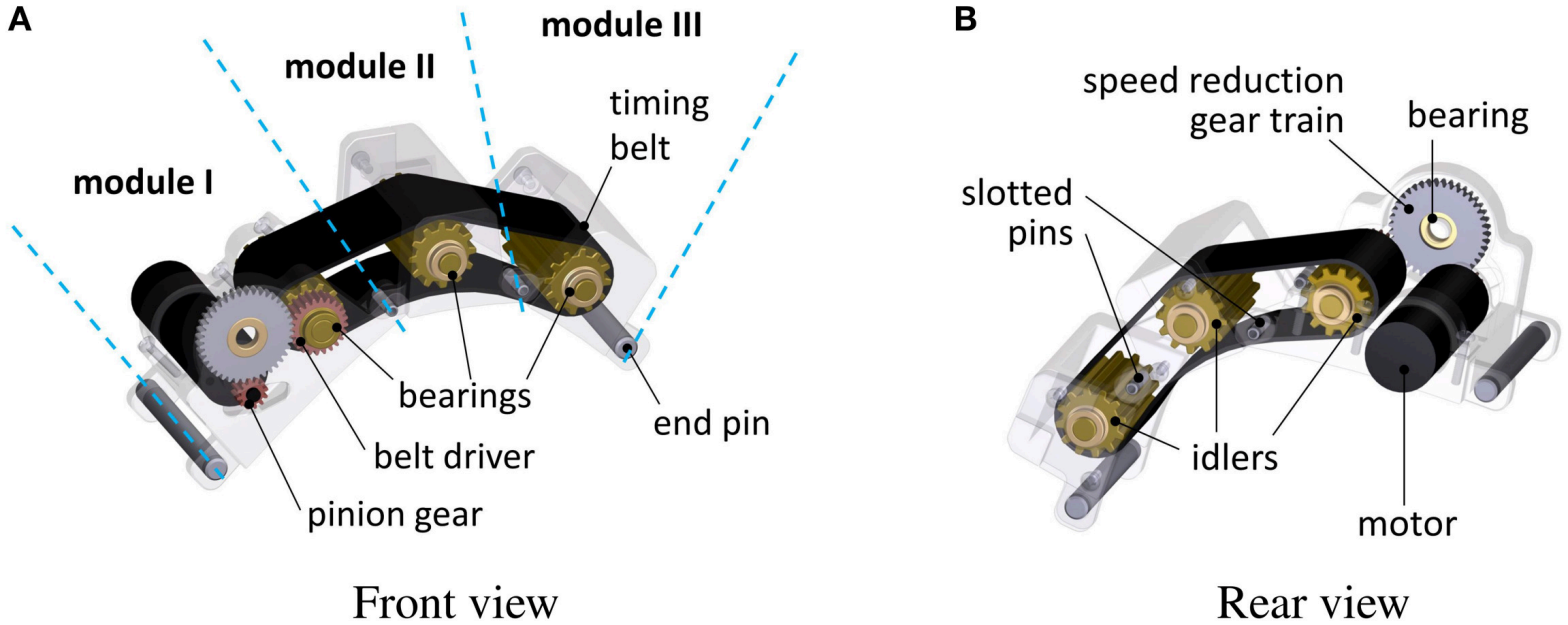
The anatomy of the wearable skin-stretcher: Front view **(A)** and Rear view **(B)**. The entire length can be adjusted by the number of Module II with respect to the subject's forearm thickness.

The anatomy of the wearable skin-stretcher is illustrated in Figure 4 (Note that we labeled subparts in Figures 4A,B for clear views). The wearable skin-stretcher mainly consisted of a timing belt that stretches the subject's skin and its driving mechanism actuated by a DC motor (1524T009SR, Faulhaber, Croglio, Switzerland) with a gear train of 2:1 reduction ratio. The timing belt was supported by cylindrical idlers. The cylindrical idlers were serrated with teeth compatible with those of the timing belt. To cope with the various forearm sizes of users, we adopted a modularized design approach as follows: the driving mechanism module (Module I), the middle idler module (Module II), and the end idler module (Module III). Therefore, the length of the skin-stretcher can be adjusted by increasing the number of Module II appropriately with respect to the user's forearm thickness.

**Figure 5:**
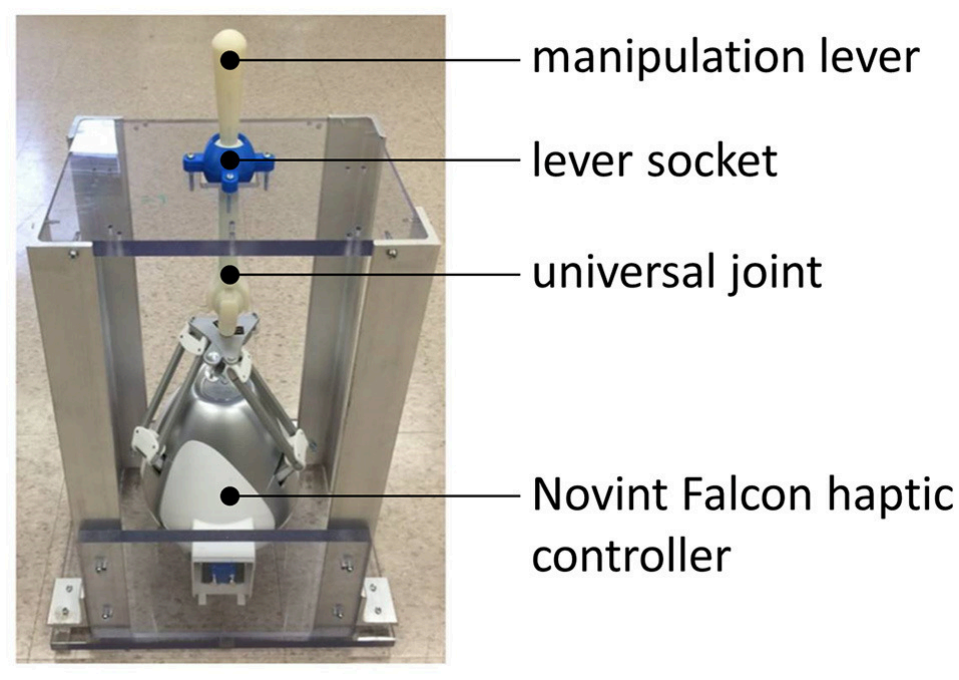
The modified 2D haptic joystick: 3D movement maps into 2D plane by adopting the universal joint and the lever socket.

Figure 5 shows the haptic joystick that was built up from the commercially-available haptic controller (Novint Falcon controller, Novint Technologies, Albuquerque, NM) and 3D printed universal joint and a manipulation lever. This haptic joystick constrains the three dimensional Falcon controllers and renders two dimensional movement. The generated force was transferred to the subject's manipulating hand by the manipulation lever.

Adopting the two developed devices enabled the subjects to receive a force feedback and a cutaneous skin stretch feedback simultaneously. Specifically, subjects could perceive the assistive force at their hand and the skin stretch at their forearm. These two additional sensory cues were expected to improve the subject's motor control by providing information about both the manipulating hand and the forearm movement, which are closely related to an individual's steering control performance. In Section 2.3, an approach to determining the direction and the intensity of these two augmented sensory feedback will be discussed in detail.

### 2.3. Approach to determining the direction and the intensity of the two augmented sensory feedbacks

**Figure 6:**
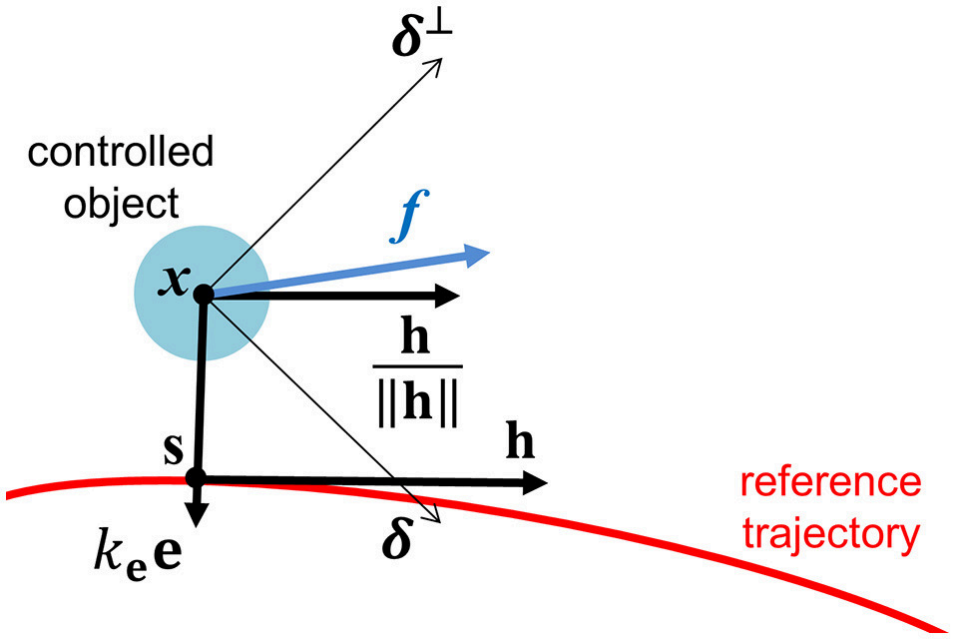
A graphical illustration of the preferred direction.

Let e be a vector pointing from the current power-wheelchair position to the closest point on γ as illustrated in Figure 6. Also, let h and *f* be a tangential vector on the shortest point and a control force generated by the rider's input, respectively. We define a vector toward a direction to where the power-wheelchair is attracted to γ as

(1) $$\delta =\text{signum}\left(f^{T}\text{h}\right)\frac{\text{h}}{\left\|\text{h}\right\|}+k_{\text{e}}\text{e}$$

where *k*_e_ is a positive constant. The direction of δ will be called *preferred direction*. Correspondingly, the orthogonal direction to δ will be called *non-preferred direction* and be denoted by δ^⊥^. By decomposing with respect to the preferred and the non-preferred directions, *f* can be expressed as the summation of two decomposed components, i.e., *f* = *f*_δ_ + *f*_δ_⊥. We can derive a control force *f*_attracted_ which makes the power-wheelchair attracted to γ by attenuating *f*_δ_⊥ component

(2) $$f_{\text{attracted}}=f_{\delta }+\kappa_{\delta^{⊥}}f_{\delta^{⊥}}$$

where aforementioned $\kappa_{\delta^{⊥}}$ is defined as a monotonically non-increasing function of ∥e∥

(3) $$\kappa_{\delta^{⊥}}(\|\text{e}\|)=\{\begin{array}{ll} 1.0, & \text{if }\|\text{e}\|\leq \frac{d}{2}-\nu \\ {\underline{\kappa }}_{\delta^{⊥}}+{\left[\frac{d/2-\|\text{e}\|}{\nu }\right]}^{2}(1-{\underline{\kappa }}_{\delta^{⊥}}), & \text{if }\frac{d}{2}-\nu <\|\text{e}\|\leq \frac{d}{2}\\ {\underline{\kappa }}_{\delta^{⊥}}, & \text{if }\|\text{e}\|>\frac{d}{2}. \end{array}$$

Namely, $\kappa_{\delta^{⊥}}$ determines how much portion of the non-preferred direction component in *f* is preserved (Bettini et al., 2004; Yoon et al., 2017). We also set a guidance zone by ν so that no guidance is provided if the deviation from γ is less than $\frac{d}{2}-\nu$, which implies the power-wheelchair is well tracking the given γ by the rider's control. The guidance zone is typically defined as a relative term to the GVF width, e.g., $\nu =0.9\times \frac{d}{2}$.

The intensity of both force feedback and skin-stretch feedback can be determined in terms of $\kappa_{\delta^{⊥}}$. By performing simple algebraic manipulation on (Equation 2), we have

(4) $$\begin{array}{l} f_{\text{attracted}}=f_{\delta }+\kappa_{\delta^{⊥}}f_{\delta^{⊥}}\\ \;=f_{\delta }+f_{\delta^{⊥}}-(1-\kappa_{\delta^{⊥}})f_{\delta^{⊥}}\\ \;=\underset{\text{originalterm}}{\underset{︸}{f}}+\;\underset{\text{guidance-related term}}{\underset{︸}{\left[(1-\kappa_{\delta^{⊥}})(-f_{\delta^{⊥}})\right]}}. \end{array}$$

**Figure 7:**
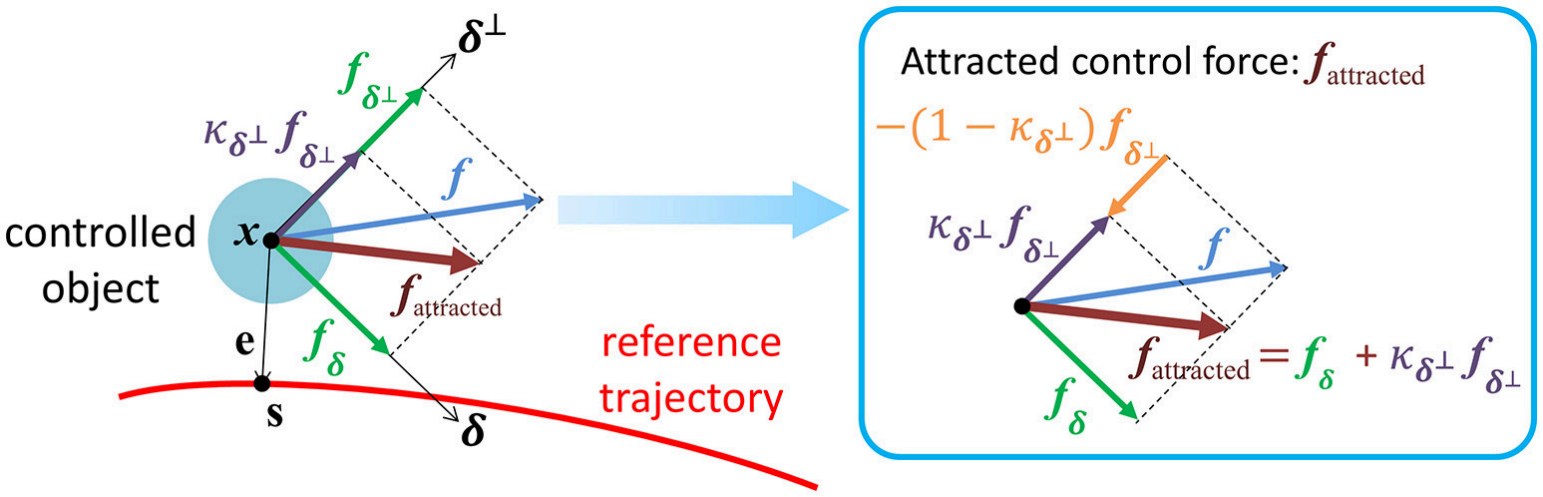
A graphical illustration of the attracted control force *f*_attracted_. The intensity of guidance is determined by 1 − κ_δ_⊥.

Equation (4) represents that $1-\kappa_{\delta^{⊥}}$ determines the intensity of guidance as illustrated in Figure 7. Therefore, let *f*_haptic_ and ω_skin_ be force feedback by haptic joystick to the rider's hand and the angular velocity of an actuator of the wearable skin-stretcher, respectively. The intensities of those two feedbacks are

(5) $$\left\|f_{\text{haptic}}\|=k_{\text{haptic}}(1-\kappa_{\delta^{⊥}})\|f_{\delta^{⊥}}\|\text{ and }|\omega_{\text{skin}}|=k_{\text{skin}}(1-\kappa_{\delta^{⊥}}\right)$$

where two positive coefficient *k*_haptic_ and *k*_skin_ can be determined by system designer's choice based on the specifications of the haptic joystick and the actuator.

The direction of force feedback *f*_*haptic*_ is straightforwardly determined with respect to the local coordinate frame of the power-wheelchair. The *y*-direction of haptic joystick (anterior-posterior direction with respect to the rider's body trunk) is set to be aligned to the current heading direction of the power-wheelchair. Therefore, *f*_*haptic*_ applied to the rider's hand will be directing to where the power-wheelchair is attracted to γ. The directions of skin-stretch feedback can be determined by checking the following condition: “At which sides of γ the power-wheelchair is running: Left or Right?” This conditions can be simply checked by

(6) $$\text{signum}(\text{h}\times f)\;=\{\begin{array}{l} +1,\text{ if the power-wheelchair is at the}\\ \text{ left side of }\gamma \\ -1,\text{ if the power-wheelchair is at the}\\ \text{ right side of }\gamma . \end{array}$$

Therefore, we can determine a skin-stretch direction to guide the rider, left-to-right or right-to-left, by rotating the actuator of the skin-stretcher clockwise/counter-clockwise. The details of our guidance algorithm also can be found in Yoon et al. (2017).

## 3. Human subject experiments

### 3.1. Subjects and experimental apparatus

**Figure 8:**
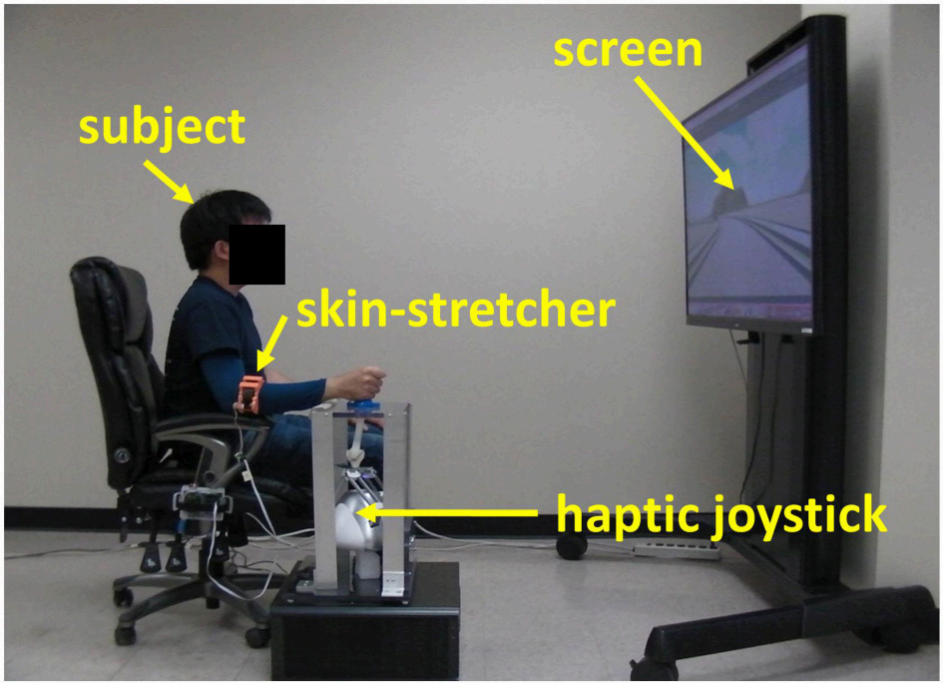
A subject is driving a virtual power-wheelchair during the experiment.

Fifteen healthy elderly adults (7 male and 8 female, age = 72.8 ± 6.6) participated in this study. None of them had former experiences with either haptic or skin-stretch devices. Subjects were seated at 1.5 m away from a 105-by-81cm (width-by-height) display as shown in Figure 8. They were instructed to drive a virtual power-wheelchair along the road with obstacles in the virtual environment where the curvature of the road varied depending on the difficulty condition. This study was carried out in accordance with the recommendations of Yeungnam University Institutional Review Board (IRB number: 7002016-A-2016-033) with written informed consent from all subjects. All subjects gave written informed consent in accordance with the Declaration of Helsinki.

### 3.2. Procedure

The experiment consisted of two sessions: a practice session and an evaluation session. During the practice session, subjects tried to familiarize themselves with the virtual power-wheelchair simulator. With the first person's view point, subjects manipulated the haptic joystick to control the virtual power-wheelchair along the road. Neither force feedback nor skin-stretch feedback was provided during the practice session. The road used in the practice session was not presented to the subjects again during the evaluation session. The practice session was limited to five minutes.

**Figure 9:**
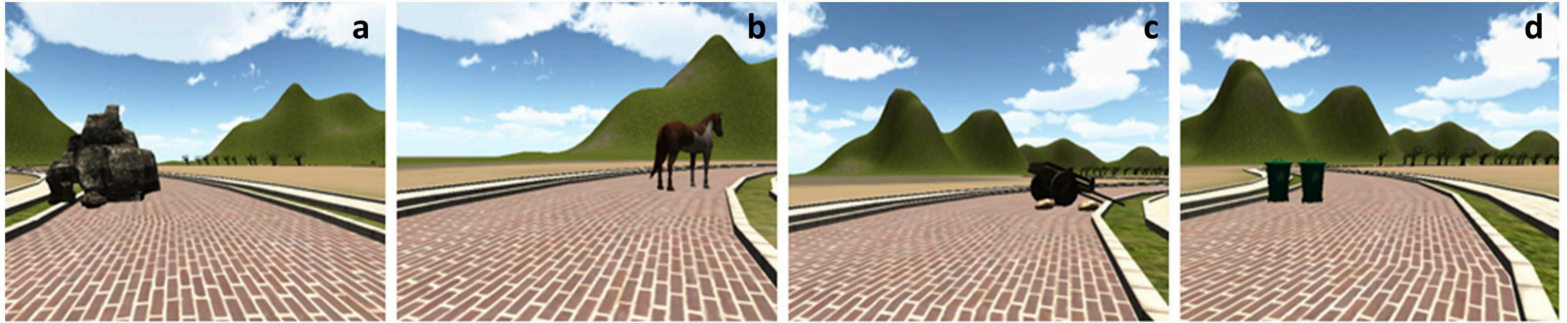
Four task scenarios with various road curvatures and obstacles: **(a)** Task1, **(b)** Task2, **(c)** Task3, and **(d)** Task4.

For the evaluation session, four task scenarios were given to subjects. Figure 9 illustrates the four task scenarios: Task1 (smooth left turn with a rock on the road), Task2 (smooth right turn with a horse on the road), Task3 (sharp right turn with a cart on the road), and Task4 (sharp left turn with trash cans on the road). In each scenario, subjects were exposed to a road with different curvatures at the point where an obstacle was placed. Subjects were instructed to pass the obstacle and reach the goal position (marked as a white finish line on the road) as fast and safe as they could without colliding to the obstacle or the road boundaries. During the evaluation session, the augmented sensory feedback for guidance was provided based on four conditions: NA (no feedback augmentation), F (force feedback augmentation by the haptic joystick), C (cutaneous skin stretch feedback augmentation by the skin-stretcher), FC (force feedback and cutaneous skin stretch feedback augmentations by both devices). Each task trial was presented with a random combination of the four task scenarios and the four sensory feedback augmentation conditions. Each trial was repeated three times; therefore, there were 4 (task scenarios) × 4 (augmented sensory feedback conditions) × 3 (repetition) = 48 total trials. Data for each subject was stored in the following format: [time stamp, *x*-position, *y*-position, heading].

### 3.3. Metrics to evaluate the subject's performance

**Figure 10:**
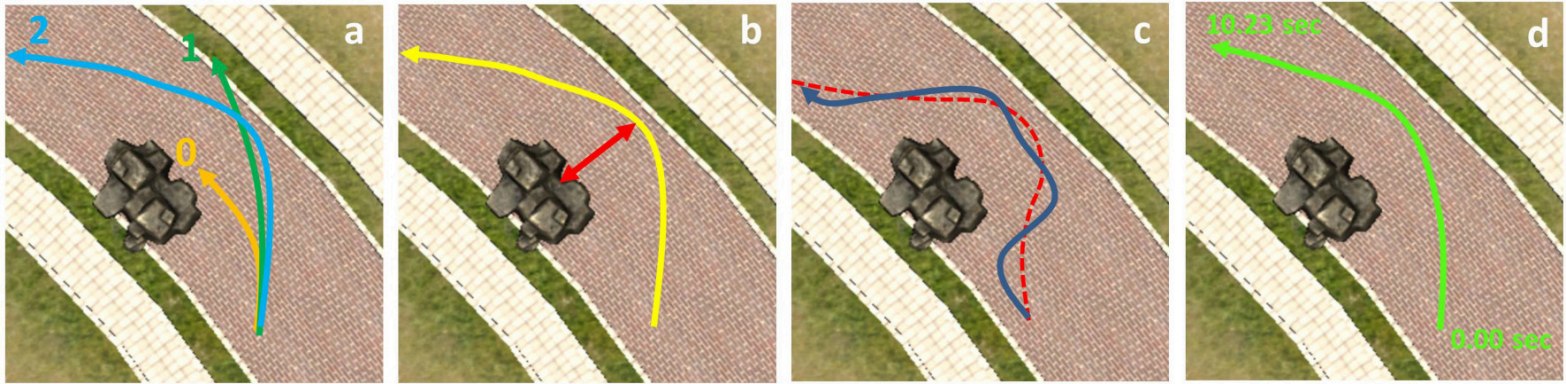
Four metrics to evaluate the subject's performance: **(a)** quality of achievement (M1), **(b)** the minimum distance to an obstacle (M2), **(c)** mean deviation from a reference trajectory (M3), and **(d)** the summation of average task completion time (M4).

For aged power-wheelchair riders, safety must be of foremost importance while driving the power-wheelchair. We were also interested in checking whether the subject's control became consistent when guidance was provided. For this reason, we adopted the following three safety-related metrics and one time-related metric to evaluate the subject's performance (see Figure 10 for illustration):

- Quality of achievement (M1) - This metric represents how successful the subjects trial was: 1 point for successfully passing an obstacle and another 1 point for reaching a goal; thus the maximum score is 2 points per each trial.
- The minimum distance to an obstacle (M2) - It measures how safely the virtual power-wheelchair passed the obstacle.
- Mean deviation from a reference trajectory (M3) - This metric quantifies how supportive the reference trajectory was.
- Summation of the average task completion time (M4) - It represents how consistently and fast the subject performed multiple trials per each task scenario.

### 3.4. Data analysis

We have two factors to consider: (i) task scenario and (ii) augmented sensory feedback. To further investigate the effects (main and interaction) of the two factors on the subjects' performance (which is represented by four metrics: M1 through M4), a two-way repeated measures analysis of variance (rANOVA) was performed on M1 through M4 (SPSS v21, Chicago, IL). Significance level was set to *p* < 0.05, and multiple pairwise comparisons were adjusted by Bonferroni correction.

## 4. Results

We present both the main and the interaction effects of two independent factors, task scenario and augmented sensory feedback, on the subjects' performance, quality of achievement (M1), minimum distance to obstacle (M2), mean deviation from reference trajectory (M3), and the summation of average task completion time (M4). Recall that we have denoted four augmented sensory feedback conditions as NA (no feedback augmentation), F (force feedback augmentation by the haptic joystick), C (cutaneous skin stretch feedback augmentation by the skin-stretcher), and FC (force feedback and cutaneous skin stretch feedback augmentations). Four task scenario conditions will be simply denoted by numbers as Task1, Task2, Task3, and Task4 in below.

### 4.1. Effects of augmented sensory feedback on subject's performance

**Figure 11:**
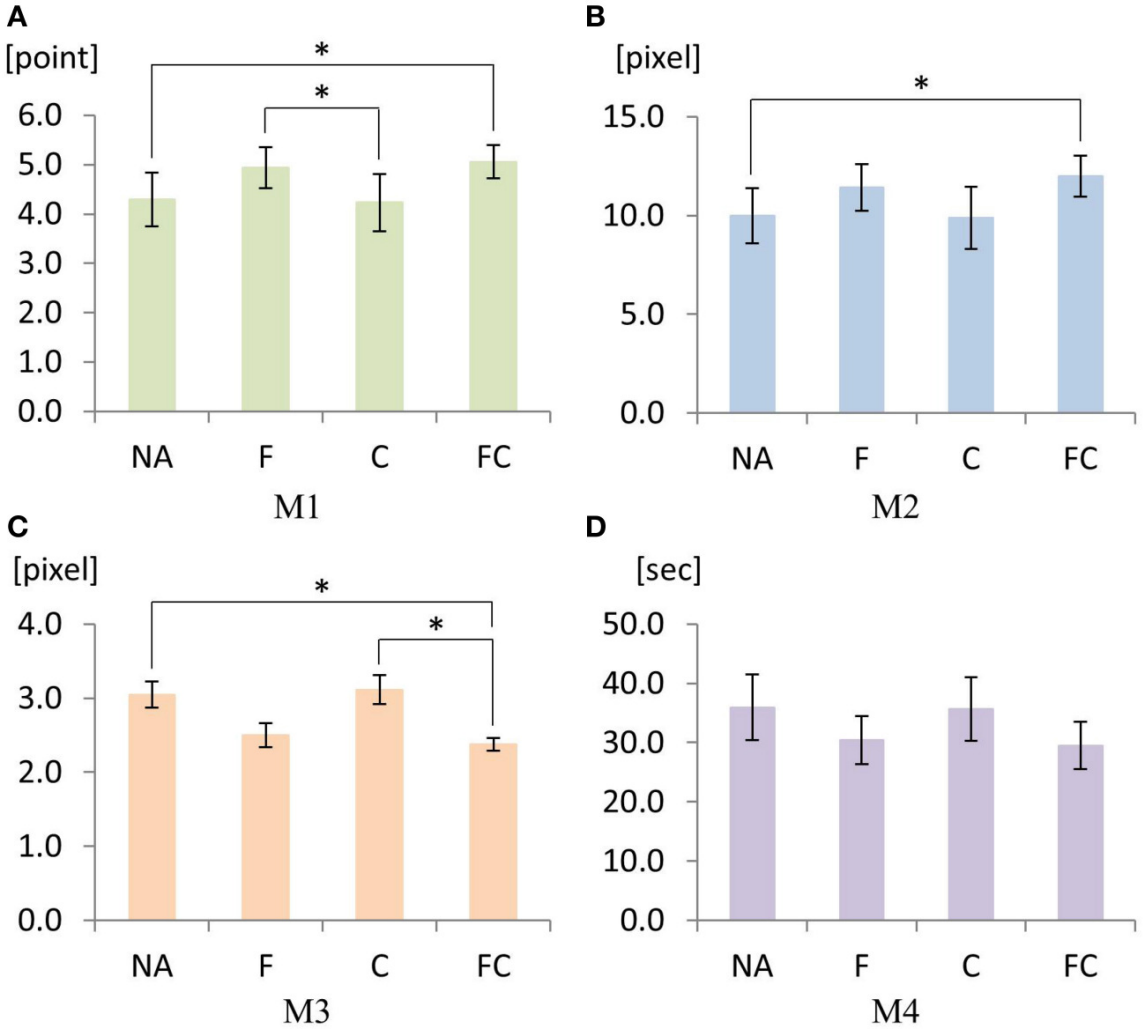
Mean values for M1 **(A)** through M4 **(D)** under four augmented sensory feedback conditions: NA (no feedback augmentation), F (force feedback augmentation by the haptic joystick), C (cutaneous skin stretch feedback augmentation by the skin-stretcher), and FC (force feedback and cutaneous skin stretch feedback augmentations). An error bar represents standard error. Significances are indicated by “_*_” for *p* < .05 resulted from Bonferroni adjusted pairwise comparisons.

First, augmented sensory feedback guidance showed a significant main effect on M1 [*p* < 0.01, *F*_(1.97, 23.63)_ = 5.95]. Pairwise comparison revealed that NA vs. FC are significantly different (*p* = 0.048), suggesting that combined sensory feedbacks for guidance significantly enhanced M1. Pairwise comparison also found that F are C significantly different (*p* = 0.028), implying that the quality of achievement under F was significantly greater than that of under C (Figure 11A).

Second, augmented sensory feedback also yielded a significant main effect on M2 [*p* = 0.01, *F*_(1.89, 22.64)_ = 5.82]. Pairwise comparison found a significant mean difference between NA vs. FC (*p* = 0.015), indicating that the minimum distance to an obstacle was significantly larger when both sensory feedbacks were applied (Figure 11B).

Third, we found a significant main effect of augmented sensory feedback on M3 [*p* = 0.001, *F*_(3, 42)_ = 7.27]. Pairwise comparison yielded two significant differences between FC vs. NA (*p* = 0.039), and FC vs. C (*p* = 0.026), indicating that the combined sensory feedbacks significantly reduced the deviation of the driving trajectory from the reference compared with no sensory augmentation or cutaneous skin-stretch only (Figure 11C).

Lastly, in contrast to the cases of the above three metrics, there was no significant main effect of the augmented sensory feedback on M4 [*p* = 0.165, *F*_(1.75, 20.98)_ = 1.99]. Even with the insignificance, it is still interesting to see that the mean value of FC was the smallest compared to the other variables (Figure 11D).

### 4.2. Effects of task scenario on subject's performance

**Figure 12:**
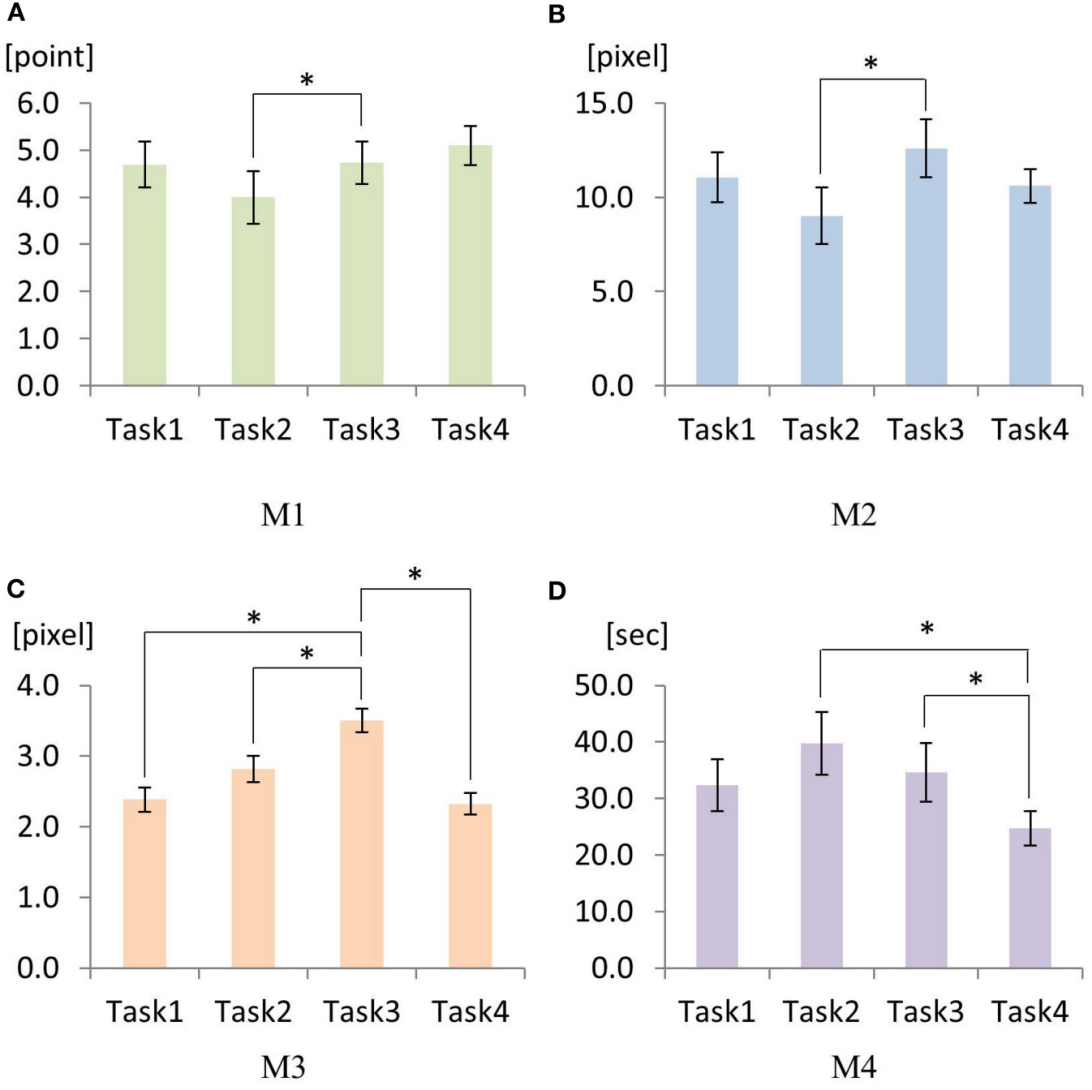
Mean values for M1 **(A)** through M4 **(D)** under four task scenarios: Task1, Task2, Task3, and Task4. An error bar represents standard error. Significances are indicated by “_*_” for *p* < 0.05 resulted from Bonferroni adjusted pairwise comparisons.

First, the rANOVA result on M1 indicated that there was a significant main effect of the task scenario [*p* < 0.01, *F*_(1.69, 20.26)_ = 6.573]. Pairwise comparison showed a significant difference between Task2 vs. Task3 (Figure 12A).

Second, M2 showed a significant main effect of the task scenario [*p* < 0.01, *F*_(1.83, 21.96)_ = 8.05]. Pairwise comparison revealed a significant difference between Task2 vs. Task3 (*p* < 0.001) (Figure 12B).

Third, a significant main effect of the task scenario was found on M3 [*p* < 0.01, *F*_(3, 42)_ = 12.84]. Pairwise comparison revealed that there were significant differences between Task3 vs. Task1 (*p* < 0.01), Task3 vs. Task2 (*p* = 0.30), and Task3 vs. Task4 (*p* < 0.01) (Figure 12C).

Lastly, there existed a significant main effect of the task scenario on M4 [*p* < 0.01, *F*_(3, 42)_ = 8.903]. Pairwise comparison showed significant differences for Task2 vs. Task4 (*p* = 0.013) and for Task3 vs. Task4 (*p* = 0.024) (Figure 12D).

### 4.3. Interaction effects of task scenario × augmented sensory feedback on subject's performance

**Figure 13:**
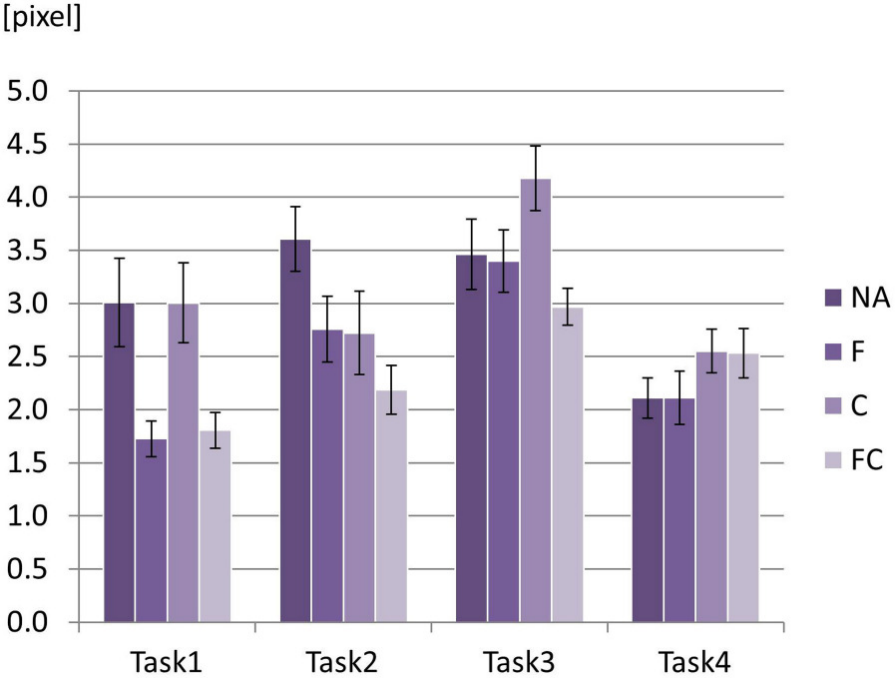
Mean values for M3 with two factors: four task scenarios × four augmented sensory feedback conditions. An error bar on the top of each graph represents standard error.

A significant interaction effect between task scenario and augmented sensory feedback was found on M3 [*p* = 0.022, *F*_(4.27, 51.20)_ = 3.06)] indicating that the effect of augmented sensory feedback on M3 was different depending on the condition of task scenario (Figure 13). Pairwise comparison revealed that the mean differences of M3 across the task scenarios became significant only when NA or C was provided. For NA condition, the significance occurred for Task2 vs Task4: 1.50 ± 0.36 (mean difference ± SE) (*p* < 0.01). For C condition, the significance happened for Task1 vs Task3: 1.18 ± 0.23 (*p* < 0.01), Task2 vs Task3: 1.46 ± 0.43 (*p* < 0.01), and Task3 vs Task4: 1.63 ± 0.35 (*p* < 0.01). For the other sensory feedback conditions (i.e., F and FC), there were no significant changes of M3.

## 5. Discussion

The results show that both task scenario and augmented sensory feedback had significant main effects on safety-related metrics i.e., M1, M2, and M3. First of all, for the augmented sensory feedback, NA and FC were significantly different for M1, M2, and M3, consistently. To be more specific, when both sensory feedbacks were simultaneously provided, the quality of driving (M1) improved, the minimum distance from the obstacle became greater with more safety margin (M2), and the deviation from the reference trajectory became smaller (M3). However, force feedback alone (F) was not enough to enhance the safety-related measures with statistical significance. Interesting observation is that a combined sensory augmentation of both force feedback and skin-stretch feedback effectively enhanced the safety-related metrics (e.g., M1, M2, and M3). This can be interpreted as follows: FC had more distinct effects on the improvement of the subjects' performance compared to the other augmented sensory feedback conditions, i.e., NA, F, and C. Even though each of F and C may not effectively enhance the safety-related metrics, the combined sensory feedback FC synergistically enhanced the safety-related metrics. This result is consistent with the findings from Shull and Damian (2015); Pan and Hur (2016); Pan et al. (2017) that skin is a good receptor; therefore, cutaneous stimulations have the great feasibilities to serve as an efficient augmented sensory feedback. Similarly, for gait and balance rehabilitation trainings, the feasibility to improve learning efficiency was attested by stimulating multiple skin locations so that the subjects can perceive different haptic stimuli more accurately (Jirattigalachote et al., 2011; Lurie et al., 2011).

Further investigation on M3 (i.e., deviation from the reference trajectory) under FC shows the feasibility of positive synergistic effect between force feedback and cutaneous skin stretch feedback. For the proposed guidance approach, the artificially-generated (software-generated) reference trajectory was invisible to the human subjects. In M3, C, and F separately didn't show any effect even though F seemed to have smaller mean value compared to C. However, the combined FC could effectively minimize the deviation of their driving trajectory from the reference, suggesting synergistic sensory augmentation from both F and C (see Figure 11C).

There were significant main effects of the task scenarios on the performances. The pairwise comparison found significant differences between Task2 and Task3 for all of the safety-related metrics (i.e., M1, M2, and M3). Task2 and Task3 have different road curvatures; the former is a smooth right turn whereas the latter is a sharp right turn. Therefore, this geometrical difference (or, difficulty) of the road was explicitly reflected by the pairwise comparison of subjects' performance. For the definition of each task, please refer to Section 3.2 where we defined four task scenarios as Task1: smooth left turn with a rock on a road, Task2: smooth right turn with a horse on a road, Task3: sharp right turn with a cart on a road, and Task4: sharp left turn with trash cans on a road.

For M1 and M2, performance of Task3 was better than the performance of Task2, suggesting that subjects performed better when the tasks were more challenging (e.g., Task3) compared to the easier task (e.g., Task 2). Similar observation was made for M4 (Task 2 vs. Task 4). One possible postulation can be provided via the relation between task performance improvement and the subject's alertness about the environment. It has been reported that people's motor performance may enhance when the motor tasks become more challenging with an appropriate amount as per their performance limit because people become more alert and cautious about the motor tasks when they encounter challenges. For example, it was reported that firefighter's functional balance enhanced significantly when they became more alert and cautious about the firefighting environment (Hur et al., 2013).

However, for M3, interesting results were found. First of all, less challenging tasks seemed to yield better performance for M3 (i.e., deviation from the reference trajectory). For example, Task1 was less challenging then Task2 since Task1 involved inward rotation (left turn) of the forearm (i.e., pronation) and Task2 involved outward rotation (right turn) of the forearm (i.e., supination) even though both Task1 and Task2 involve the same radii of curvature. It has been reported that control of inward arm movement is more dexterous than the control of outward arm movement. For example, forehand stroke is more accurate and powerful than backhand stroke in tennis or table tennis (Mavvidis et al., 2010). Also, it was reported that torque generation of the forearm in inward direction is more efficient and precise when outward direction (Seo et al., 2007, 2008). Also, please note that there was no effect of hand dominance since all subjects were right-handed. Similarly, trajectory deviated more for Task3 compared to Task1 and Task2, since Task3 is more challenging. For Task4, significant reduction in deviation was found even though Task4 involves sharp turn to the left direction. This can also be explained by the inward rotation of the forearm.

Please recall that there was a significant interaction effect between the task scenario and the augmented sensory feedback on M3 (Figure 13). The interaction effect is mainly due to the results that M3 was affected by the task scenario only when NA or C was applied as a sensory feedback condition. This suggests that NA or C does not affect the performance of M3; hence, the task characteristics (e.g., radius and direction of curvature) are directly reflected in the performance of M3. This also indicates that F or FC condition dominates the performance of M3 such that the task characteristics are not directly reflected in the performance of M3.

If we consider C and F as independent sensory augmentation modalities, the data in Figures 11–13 indicate that F dominates C and that C itself has no effect on the performance. However, when both F and C are combined, we could observe the synergistic effect of both F and C such that the overall performance (i.e., M1, M2, M3, and M4) became maximized with FC (Figures 11, 12). This synergistic behavior of FC can be explained with the effect of stochastic resonance for sensorimotor control systems. Stochastic resonance is a phenomenon in which the addition of unperceivable noise (e.g., vibrotactile noise) to a weak signal maximizes the detection and transmission of the weak signal through the neuronal network (Enders et al., 2013). When subsensory threshold vibration is applied to the skin, people do not perceive the vibration. Also, when a weak sensory signal is applied to the skin, people may or may not detect the signal, and the motor performance based on the weak signal would be suboptimal. However, when the weak sensory signal is combined with the subsensory threshold vibration, the detection of the signal enhances, and the motor performance based on the enhanced sensation is maximized (Hur et al., 2014; Seo et al., 2014).

In a broad sense, both cutaneous (or skin-stretch in this study) sensory feedback and force feedback can be regarded as a subset of haptic guidance. The difference lies in how human perceives each of the two augmented sensory feedbacks. Skin-stretch sensory feedback is perceived by Ruffini corpuscle and Meissner corpuscle, the mechanoreceptors underneath the skin, which are sensitive to skin stretch and skin slip, respectively (Johansson and Westling, 1987). In contrast, force feedback to the hand is perceived by Merkel corpuscle, the mechanorecpetor underneath the skin, which is sensitive to sustained pressure (Johansson and Westling, 1987). Force feedback is also perceived by Golgi tendon organ, a proprioceptor at each joint, that is sensitive to muscle tension and pull of tendons (Stephens et al., 1975). Therefore, skin-stretch feedback and force feedback are highly likely to have different mechanisms for sensing and processing sensory signals, and thus, the effects of C and F in the performance metrics (i.e., M1, M2, M3, and M4) seem to differ (Figures 11, 12). However, it's not well known how two sensory feedback signals are fused and processed to produce the synergistic effects, and thus deserves further investigations.

Even though it's not the scope of this study, it will be interesting to look into the effect of multiple modalities on the performance indices. It was reported that different modalities have different effects on the performance indices (Wang et al., 2011; Ronsse et al., 2011). For example, auditory alertness helped enhance the driving performance whereas visual feedback alone did not (Wang et al., 2011). Even though multiple modalities can be useful in enhancing the driving performance, haptic modality can have more direct effects on the improvement since visual or auditory modalities are already heavily used while conducting the tasks (Scott and Gray, 2008; Wang et al., 2011; Yoon et al., 2017). In our study, other modalities, e.g., vision or audition, were not included than haptic modalities. As reported in the result section, force feedback and skin stretch feedback have different effects in enhancing the performance indices even though both synergistically enhanced the performance. It will be interesting to investigate how other modalities, e.g., vision or audition, can influence the synergistic behavior. In addition, it will be worth examining the way both of force feedback and skin stretch feedback are integrated to maximize the enhancement.

In design perspectives, the future usage of the custom-developed wearable skin-stretcher is promising. Considering guidance hypothesis, we know that the augmented sensory devices should be worn persistently to improve the activities in daily living for users (Sigrist et al., 2013). The features of our skin stretchers – a small size, arm-band type, and wirelessly-controlled – show us that it can cope with the needs to assist the users in daily living. Existing research have reported the effectiveness of haptic guidance for power-wheelchair riders (Marchal-Crespo et al., 2010a,b; Morère et al., 2015; Foley et al., 2016). Therefore, in practical situation, we also expect that the proposed method support the power wheelchair riders successfully by combining with existing machine vision and sensor systems, such as introduced in How et al. (2013) and Simpson et al. (2004), to generate an artificial reference path based on the surroundings.

Although the balances in both age and gender were concerned when the subjects were recruited, the limitation of this study is that the subjects were not actual power wheelchair riders. Therefore, there exists a possibility of that the result might vary; however, we remained checking this possibility as future work. Also, the subjects performance while riding a practical system should be investigated as well.

## 6. Conclusion

In this study, we developed two augmented sensory feedback devices, a wearable skin-stretcher and a 2D haptic joystick, and provided elderly subjects with additional sensory cues via cutaneous skin stretch feedback and force feedback at their forearm and hand, respectively. The experiments were performed with four task scenarios in which the subjects were instructed to drive a virtual power-wheelchair along roads of various curvatures where obstacles were placed. The subjects' task performances were evaluated based on the following four metrics: quality of achievement (M1), minimum distance to obstacle (M2), mean deviation from a reference trajectory (M3), and the summation of average task completion time (M4). The results showed that M1, M2, and M3 were improved, compared to no feedback augmentation condition, when both a force feedback and a cutaneous skin stretch feedback were provided simultaneously. Especially, for M3, the cutaneous skin stretch itself did not seem to encourage the performance improvement; in contrast, the improvement was observed when it was combined with a force feedback. These findings substantiated that there existed the positive synergistic effects on elderly power-wheelchair riders' safety metrics when the two augmented sensory feedbacks were combined.

Future work should be followed to examine the efficacy of the proposed sensory augmentations–that is, the cutaneous skin stretch feedback on the forearm and the force feedback–for different tasks, e.g., hand writing, drawing shapes, learning motions in sports, will be of our interest. Next, further investigation should be conducted in physiological perspectives “why a cutaneous skin stretch and a force feedback encourages the performance improvement when they are combined in?” Accordingly, we should also check whether the encouraged improvement depends on stimulated locations–both sensory augmentations should be applied on ipsilateral or contralateral side, or whether the improvement will be consistent when one of those feedbacks works as a disturbance. Lastly, the various combinations of augmented sensory feedback should be considered to compensate or overcome degraded task performance in activities of daily living such as turning a door knob, spooning, waking up/down stairs, etc.

## Author contributions

HY and PH have contributed to a theoretical concept, the design of the experiment, performing experiments, data analysis, interpretation, and drafting the manuscript. NA has contributed to design of the wearable skin-stretcher.

### Conflict of interest statement

The authors declare that the research was conducted in the absence of any commercial or financial relationships that could be construed as a potential conflict of interest.
